# Perceptions of Patients, Caregivers, and Healthcare Professionals toward Telemedicine Use for Cognitive and Movement Disorders in the Aegean Islands, Greece: A Pilot Study of the SI4CARE European Project

**DOI:** 10.3390/geriatrics9010003

**Published:** 2023-12-26

**Authors:** Efthalia Angelopoulou, Dionysia Kontaxopoulou, Stella Fragkiadaki, Evangelia Stanitsa, Dimosthenis Pavlou, John Papatriantafyllou, Christos Koros, Vlado Dimovski, Darja Šemrov, Sokratis G. Papageorgiou

**Affiliations:** 1 1st Department of Neurology, Aiginition University Hospital, Vasilissis Sofias Street 72-74, 11528 Athens, Greece; d.kontaxopoulou@hotmail.com (D.K.); st.fragkiadaki@gmail.com (S.F.); eva.st.92@gmail.com (E.S.); jpapatriantafyllou@gmail.com (J.P.); chkoros@gmail.com (C.K.); sokpapa@med.uoa.gr (S.G.P.); 2 School of Topography and Geoinformatics, University of West Attica, Ag. Spyridonos Str., 12243 Aigalew, Greece; dpavlou@central.ntua.gr; 3 School of Economics and Business, University of Ljubljana, Kardeljeva ploščad 17, 1000 Ljubljana, Slovenia; 4 Faculty of Civic and Geodetic Engineering, University of Ljubljana, Jamova Cesta 2, 1000 Ljubljana, Slovenia

**Keywords:** telemedicine, teleneurology, Alzheimer’s disease, Parkinson’s disease, dementia, cognitive impairment, movement disorders, telehealth, telecare

## Abstract

Background: Patients with neurodegenerative diseases who live in remote areas often have limited access to specialized healthcare, and telemedicine represents a useful solution. The aim of this study was to investigate the perceptions toward the use of a specialized-tertiary telemedicine service of patients with cognitive and movement disorders, caregivers, and local healthcare professionals (HPs) in the Aegean Islands. Methods: Data were derived from the “Specialized Outpatient Clinic of Memory, Dementia and Parkinson’s disease through the National Telemedicine Network”, March 2021–March 2023. The survey included 10 questions (5-point Likert scale). Results: We received 64 questionnaires (25 patients, 18 caregivers, 21 HPs). Most participants positively perceived all aspects of telemedicine, including comfort (mean ± standard deviation: patients 4.5 ± 0.9, caregivers: 4.8 ± 0.5, HPs: 4.6 ± 0.7), access to specialized care (4.7 ± 0.6, 4.7 ± 0.5, 4.9 ± 0.4), number of transportations (4.6 ± 0.8, 4.6 ± 0.9, 4.8 ± 0.5), adequacy of follow-up (4.6 ± 0.7, 4.4 ± 0.8, 4.2 ± 0.7), future telemedicine selection (4.8 ± 0.4, 4.8 ± 0.4, 4.6 ± 0.6), perceived reliable medical assessment (4.7 ± 0.5, 4.6 ± 0.6, 4.3 ± 0.6), information delivery (4.7 ± 0.6, 4.6 ± 0.5, 4.4 ± 0.9), health status improvement (4.6 ± 0.7, 4.6 ± 0.6, 4.0 ± 0.7), cost (4.6 ± 1, 4.6 ± 1, 5.0 ± 0.2), and general satisfaction (4.8 ± 0.4, 4.7 ± 0.5, 4.5 ± 0.6). The commonest recommendations were more frequent visits, medical specialties, and dissemination of information. Conclusions: The positive perception of participants highlights the value of telemedicine for specialized healthcare for neurodegenerative disorders, especially in remote areas.

## 1. Introduction

Due to the growing ageing population, the prevalence of several age-related neurological diseases will continuously increase, resulting in a growing burden on healthcare systems. Neurodegenerative disorders comprise a clinically heterogeneous group of chronic diseases characterized by the progressive and selective loss of neurons, accompanied by functional impairment. Alzheimer’s disease is the most common neurodegenerative disorder and the leading cause of dementia, followed by Parkinson’s disease, which is the most frequent movement disorder. Although there is no definite cure for neurodegenerative disorders, early diagnosis and appropriate management have been associated with improved quality of life [1]. Detailed neurological and neuropsychological assessments are often required for diagnosis, especially in atypical cases. However, patients who live in remote areas often have limited access to specialized healthcare because of the long waiting times and high travel burden for neurological and neuropsychological assessment, the lack of specialized healthcare professionals in rural areas, and the inadequate knowledge of general practitioners to diagnose and treat these diseases [2]. Loss of autonomy, mobility issues, other comorbidities, and difficulties in navigating the complicated healthcare system create additional challenges. Compared to urban regions, the prescription of inappropriate medications and ambulatory care-sensitive hospitalizations of patients with dementia are higher in rural areas, and caregivers display more difficulties in the management of behavioral symptoms [3].

Telemedicine, referring to remote diagnosis and treatment through information and communications technology (ICT), represents a useful tool for bridging this widening gap [4]. During the COVID-19 pandemic, the need for remote healthcare was further emphasized, particularly for vulnerable individuals with chronic disorders, including those with neurodegenerative diseases [2,5]. Several studies have demonstrated that neurological and neuropsychological assessments via videoconferencing are reliable for cognitive and movement disorders compared to in-person evaluation [6,7]. Telemedicine has been shown to improve the quality of care of patients with dementia, Parkinson’s disease, and other chronic neurological conditions by facilitating access to early diagnosis and specialized, personalized, and holistic care, reducing unnecessary transportations and cost, and allowing for an interdisciplinary treatment approach regardless of geographical location [2,7]. 

Greece has more than 100 inhabited islands, most of which are located in the Aegean Sea. The distance between the port of Piraeus in Attica and the Aegean Islands ranges approximately from 50 km (Aigina) to 550 km (Kastellorizo). Medical centers in many of these regions are understaffed, and patients often have to travel long distances to reach specialized medical assessment and care, while travel restrictions due to weather conditions result in additional delays. There is no neurologist, psychiatrist, or neuropsychologist in most of those islands, and inhabitants mainly receive primary care from general practitioners via regional Health Centers. During the winter months, travel restrictions further limit transportation due to bad weather conditions. This geographical peculiarity highlights the value of telemedicine and its potential to improve accessibility to specialized care. Among several efforts, the National Telemedicine Network (NTN), which has been developed by the Second Regional Healthcare Administration of Piraeus and Aegean Islands in 2016, constitutes the most integrated telemedicine application in Greece. NTN includes at least 57 physician–patient telemedicine stations in regional health centers of the Aegean Islands and 13 physician–consultant telemedicine stations in general hospitals. It aims to provide remote synchronous medical support from various specialties (dermatology, endocrinology, oncology, neurology, psychiatry, etc.) to patients living in the Aegean Islands, thereby overcoming geographical barriers. 

The Neurology Department of Aiginition University Hospital in Athens, in collaboration with the Second Regional Healthcare Administration of Piraeus and Aegean Islands, had the initiative to create the “Specialized Outpatient Clinic of Memory, Dementia and Parkinson’s disease through the National Telemedicine Network”. Via this newly designed telemedicine unit provided by a tertiary care facility, which started in March of 2021, patients with cognitive or movement disorders living in the Aegean Islands covered by the NTN are examined by specialized healthcare professionals of Aiginition University Hospital with the aim of a more accurate and earlier diagnosis, as well as appropriate treatment. 

Given the growing integration of telemedicine into routine clinical practice, it is important to identify the perceptions and satisfaction of patients, caregivers, and healthcare professionals with its use in order to improve the related services and optimize patient outcomes. Patient-centered care, which respects and responds to patients’ needs, preferences, and values, is a high priority in contemporary healthcare delivery, and user satisfaction is considered an important indicator of the successful use and adoption of telemedicine [8]. It has been demonstrated that satisfied patients display better adherence to medical instructions, leading to improved clinical outcomes, shorter hospitalization times, and more rapid recovery [8]. 

Most studies have indicated positive perceptions toward the use of telemedicine among patients with dementia and their caregivers [9,10,11,12,13,14,15], as well as among patients with Parkinson’s disease [7]. A recent scoping review aiming to describe the effectiveness and user experience of using telemedicine services for individuals with dementia and their caregivers during the COVID-19 pandemic demonstrated that overall, participants perceive this mode of care delivery positively [12]. In particular, it has been indicated that about two-thirds of caregivers of patients with dementia would be willing to use telemedicine for monitoring, and telemedicine was actually preferred if given as an option [16]. Another study in a public hospital demonstrated that about 98% of patients with dementia and caregivers were satisfied with telemedicine, avoiding long waiting times in crowded medical waiting rooms [13]. Moreover, dementia caregivers were highly satisfied with support through videoconferencing, given the travel time needed for accessing in-person services combined with their limited time because of caregiver responsibilities [17]. A study in southern Italy indicated that nine in ten patients with frontotemporal dementia and their caregivers were also satisfied with remote neurological interviews and would be interested in continuing to use this means of care [18]. Another study exploring the feasibility of implementing a telemedicine memory clinic for follow-up in a rural community demonstrated that more than 90% of patients and physicians would be willing to utilize videoconferencing in the future [19]. In that study, the vast majority of physicians also felt that remote visits provided adequate information to aid in the process of clinical decision-making, and this telemedicine service contributed to timely access to specialized care, lower rates of cancelled visits, and improved follow-up [19]. In a study of residential care veterans, participants also showed high satisfaction with telemedicine experiences and would like to receive healthcare via telemedicine again in the future [20]. 

However, relevant evidence on the perceptions of healthcare professionals is scant, and the perceptions of all three participant types (patients, caregivers, and healthcare professionals) with telemedicine for neurodegenerative diseases has not been concurrently and systematically investigated, especially in the case of remote services provided by tertiary academic centers [9,10,11]. Furthermore, literature evidence on patients’, caregivers’, and healthcare professionals’ recommendations for the improvement of telemedicine services for cognitive and movement disorders is lacking. Little is known about which factors may affect perceptions toward the use of telemedicine for these diseases. In this regard, a study aiming to describe the feasibility, satisfaction, and utilization of a video-based in-home support program for dementia care demonstrated that caregivers of patients with more advanced stages of dementia were more likely to mention that privacy was intruded by video recordings, while other factors, including caregiver gender, age, education, rural status, and dyadic relationships, were not related to ratings of ease of use, acceptability, or intervention utilization [9]. Another study among informal caregivers of patients with dementia indicated that women were more likely to have at least some knowledge about technology for caregiving, whereas men were more likely to pay more for these technologies, compared to women [21]. Female Chinese caregivers of people with dementia were also more receptive of technology compared to men [22]. However, evidence regarding the factors associated with the perceptions of participants particularly toward neurological and neuropsychological assessment through videoconferencing performed by a specialized tertiary center is scant.

Furthermore, in the Greek population, it is unclear if telemedicine is accepted by the recipients, and the perceptions of end-users have not been thoroughly investigated. Since geographical location may affect users’ satisfaction with telemedicine [23], it is important to clarify the perceptions of individuals specifically in the population of the Aegean Islands. 

The aim of this study was to investigate for the first time the perceptions of patients with cognitive and movement disorders, their caregivers, and healthcare professionals in the Aegean Islands of Greece toward the use of a specialized telemedicine service provided by an academic center during a 2-year period. This pilot study is part of the European Project SI4CARE (Social Innovation for integrated health CARE of ageing population in ADRION Regions), which aims to enhance cooperation among healthcare services for the effective application of social innovation in the health and social care of the elderly in the Adriatic-Ionian area [5].

## 2. Materials and Methods

### 2.1. Study Design and Participants

Data were derived from the “Specialized Outpatient Clinic of Memory, Dementia and Parkinson’s disease through the National Telemedicine Network” during the period March 2021–March 2023. The NTN was developed by the Second Regional Healthcare Administration of Piraeus and Aegean Islands in 2016. The NTN includes at least 57 physician–patient telemedicine stations in regional health centers of the Aegean Islands and 13 physician–consultant telemedicine stations in general hospitals. It aims to provide remote synchronous medical support from various specialties (dermatology, endocrinology, oncology, neurology, psychiatry, etc.) to patients living in the Aegean Islands, thereby overcoming geographical barriers. The main elements of the NTN are the physician–patient and physician–consultant telemedicine stations, the HelpDesk at the Second Regional Healthcare Administration of Piraeus and Aegean Islands, and the Data Center at the General Secretariat of Information Systems for Public Administration.

Through this telemedicine unit, specialized healthcare professionals from Aiginition University Hospital (two neurologists, one psychiatrist, two neuropsychologists) with experience in cognitive and movement disorders used telemedicine to examine patients living in the Aegean Islands of Greece covered by the NTN who had cognitive or motor complains, with or without a preexisting diagnosis of a cognitive or movement disorder. Participants were above the age of 18 years old and willing to be examined by specialized neurologists, neuropsychologists, and psychiatrists through telemedicine. No specific exclusion criteria were set for the individuals to be assessed by our telemedicine team via this service. Familiarity with technology and digital literacy were not required for patients and caregivers, since the local healthcare professional and the NTN HelpDesk coordinated and facilitated the whole process of telemedicine assessment. 

In order to inform local physicians about our service, two online meetings were initially conducted. The telemedicine visits were scheduled via the NTN HelpDesk upon referral from the general practitioner who had initially assessed the patient and provided a brief medical history to the telemedicine team. There were no formal referral criteria, but, during the two initial online meetings, emphasis was placed on atypical cases with uncertain diagnosis or difficult management, given that the service was provided by an academic center. The patients were evaluated in a private room of the health center or peripheral hospital of each island, in a synchronous (real-time) manner, via videoconferencing with high-resolution cameras and screens, via which the patient and physician could view life-sized images of each other on the screen in actual size. The videoconferencing was performed via the telemedicine software provided by NTN. No specific medical equipment, such as vital sign monitoring, was required for our telemedicine service. The referral (local) physician coordinated the whole process of arranging and conducting the telemedicine examination at the peripheral health center or general hospital on the islands, with the assistance of the HelpDesk of the NTN. During the telemedicine examination, which lasted approximately an hour, a local healthcare professional (general practitioner, rural doctor, nurse, psychologist, or social worker) of the health center or peripheral hospital as well as the patient’s family caregiver were also usually present. Rhodes was the only island on which a general neurologist or neurology resident was present during the telemedicine examination, while a general practitioner, rural doctor, or other healthcare professional was present during telemedicine visits on the other islands. Telemedicine visits were conducted through the National Public Administration Network “Syzefxis”, which ensured the security of personal and medical data.

The telemedicine visit included medical history taking, a focused neurological examination, and a neuropsychological assessment adapted to remote settings. The neurological and neuropsychological assessment parts were selected by our team based on a literature review and the implementation guide of the American Academy of Neurology (AAN) [24,25] in combination with our previous experience examining patients with neurodegenerative diseases during the COVID-19 pandemic via telemedicine (through videoconferencing) at the “Specialized Outpatient Clinic for Memory, Cognitive disorders and Rare forms of Dementia” of the Neurology Department of the Aiginition University Hospital. Neurological examination included gait assessment, examination of cranial nerves focusing on eye movements, motor evaluation for tremor, bradykinesia, and some components of muscle strength and coordination. It was conducted by the telemedicine medical team with the assistance of remote physicians if needed. The caregivers were also interviewed to validate or provide additional relevant information. The Unified Parkinson’s Disease Rating Scale (UPDRS) part III was also administered (except for the parts evaluating rigidity and postural instability) when parkinsonian symptoms or signs were identified. Detailed neuropsychological testing included the assessment of memory, attention, language, and praxis (Mini Mental State Examination (MMSE), Hopkins Verbal Learning Test–Revised (HVLT-R), Clock Drawing Test, Forward and Reversed Digit Span, phonemic and semantic fluency, apraxia testing, Trail Making Test A and B (verbal adaptation)). Neuropsychiatric evaluation of behavioral symptoms and functional assessment were conducted via the short version of the Informant Questionnaire on Cognitive Decline in the Elderly (IQCODE), Instrumental Activities of Daily Living (IADL), Neuropsychiatric Inventory (NPI), and Geriatric Depression Scale (GDS). Upon completion of the assessment, the telemedicine team conferred and informed the patient, caregiver, and remote healthcare professional about the consensus diagnosis, referral for neuroimaging and laboratory exams if needed, treatment recommendations, and follow-up plan. At the end of the telemedicine visit, the telemedicine medical team asked patients, their caregivers, and the healthcare professionals who were present during the exam to complete a questionnaire on their satisfaction with the telemedicine visit. 

For the assessment of perceptions toward the use of our telemedicine service, study participants were (i) patients who live in the Aegean Islands and were examined by our team for cognitive or motor complains, with or without a known cognitive or movement disorder, via the “Specialized Outpatient Clinic of Memory, Dementia and Parkinson’s disease through the National Telemedicine Network”; (ii) their family caregivers; and (iii) local healthcare professionals working in the Aegean Islands who were present during the telemedicine visit. Included participants were above the age of 18 years and willing to participate in the study and complete the provided questionnaire, while those unable to understand, read, or write in Greek were excluded. No other specific exclusion criteria were set. Since a follow-up visit might affect participants’ perceptions because of greater familiarity, participants were asked to fill in the questionnaires after the initial telemedicine visit. Patients with up to early stages of dementia, defined as having a score of Mini Mental Status Examination (MMSE) ≥ 20, were included for the completion and analysis of questionnaires. 

### 2.2. Questionnaire Development and Data Collection

Some of the most common questionnaires used in telemedicine include the Telehealth Usability Questionnaire (TUQ), Telemedicine Satisfaction Questionnaire (TSQ), and the Service User Technology Acceptability Questionnaire (SUTAQ) [26]. These questionnaires include items regarding the use of technological equipment, such as “I do not need assistance while using the system” [27], “It was easy to learn to use the system”, and “This system is able to do everything I would want it to be able to do” [28], as well as questions regarding the need for visiting general practitioners, such as “The kit I received has saved me time in that I did not have to visit my general practitioner clinic” [29]. This kind of questions are not applicable for our service, since the telemedicine examination in our study was performed via telemedicine stations at local health centers on the islands, where there was always a local healthcare provider, such as a general practitioner, who managed and facilitated the whole process of the remote assessment. In addition, many of the commonly used questionnaires on telemedicine do not include questions specifically about access to specialized care, an aspect which we would like to additionally examine in our study. For these reasons, we decided to develop a new questionnaire that would be more appropriate for the type of telemedicine service provided by our department. 

The questionnaire was developed by the telemedicine team (neurologist, psychiatrist, and neuropsychologist) based on a literature review on telemedicine, other relative questionnaires, and our previous experience, as previously described [30]. Our aim was to identify the perceptions of the participants toward the use of our telemedicine service, covering the basic aspects of telemedicine and focusing on specialized care [26]. A preliminary draft of the questionnaire was designed, and input from digital health experts was also obtained for ascertaining face validity. The questionnaire was also sent to five responders for pilot testing in order to identify confusing questions or items needing clarification. A reliability test of internal consistency was used for the data that were analyzed. Cronbach’s alpha was 0.84 for the 10-item scale, which constitutes an indicator of high reliability. 

At the beginning of the questionnaire, the aims of the study were shared, highlighting that we are referring to their telemedicine examination through the “Specialized Outpatient Clinic of Memory, Dementia and Parkinson’s disease through the National Telemedicine Network”. The first part of our questionnaire consisted of demographic characteristics, including age in years, sex (male, female), marital status (single, married, divorced, widowed), highest educational attainment (primary, secondary, tertiary, master’s or doctoral degree), island of residency, and reason for referral (cognitive or movement disorder). The second part was the survey section, which included 10 questions, which were the same for each of the three participant categories (patient, caregiver, healthcare professional). We assessed participants’ perceptions of 10 telemedicine aspects (access to specialized healthcare, convenience, number of transportations, cost, patient–physician communication, reliability of medical assessment, adequacy of follow-up, health improvement, future selection of telemedicine, overall satisfaction). At the end of the questionnaire, there was an open-ended field for recommendations for the improvement of the telemedicine service. 

For the rating of responses, a 5-point Likert scale was used: 1 (not at all), 2 (a little), 3 (neutral), 4 (very much), and 5 (extremely). Responses were subsequently divided into two subsections: the first including 1, 2, and 3 (negative to neutral), and the second including 4 and 5 (positive). 

Informed consent was obtained from the participants. The questionnaire was anonymously filled. No personal identifying information was documented, ensuring the anonymity of the surveys for data analysis. The completion of the questionnaire required approximately 5–10 min.

### 2.3. Data Analysis

Data were first analyzed with descriptive statistics. For categorical variables, absolute and percentage frequencies were used. For continuous variables, means and standard deviations were calculated. Participants’ perceptions toward telemedicine use were evaluated by measuring the frequency and percentage of each item, which is consistent with relevant studies [31,32]. Means and standard deviations (SDs), as well as medians and interquartile ranges (IQRs) of scores in each question and for each participant type were also computed. In addition, the total sum of all questions was calculated. Kruskal–Wallis H was used in order to explore the possible differences in scores among the three subgroups (healthcare professionals, patients, and caregivers). As a neurologist or neurology resident was present during telemedicine visits on Rhodes and not the other islands, two subgroups were also created based on this difference (residency on Rhodes compared to non-residency on Rhodes). Responses to questions were compared based on sex, reason for referral (cognitive or movement disorder), and island of residency (residency in Rhodes or not) using the Mann–Whitney *U* test, since non-parametric tests are usually recommended for Likert scale data [33]. Statistical significance level was set at ≤0.05. IBM SPSS Statistics 25 was used for the analysis. 

## 3. Results

### 3.1. Characteristics of Study Participants

During the study period, 119 (68 first, 51 follow-up visits) telemedicine examinations were conducted. The mean age of patients was 68 (±11.4) years, and almost two-thirds were women (62%) and married (62%). The mean number of years in education was 10 (±4.6). Approximately half of the telemedicine visits were on Rhodes (54.4%), followed by Ios, Kythira, Amorgos, Tinos, Paros, Astypalaia, Kimolos, Syros, Oinousses, Agios Efstratios, Folegandros, Kalymnos, Kos, Patmos, and Samos. The most common reasons for referral were cognitive disorders (84%). One patient was examined because of a primary complaint of trigeminal neuralgia and additional symptoms of mild memory impairment (Table 1).

In total, we received 64 questionnaires (25 patients, 18 caregivers, 21 healthcare professionals, Table 2). For patients and healthcare professionals, the response rate was 37% (25/68) and 31% (21/68), respectively. In total, the island with the highest participation regarding the completion of satisfaction questionnaires was Rhodes (73%), followed by Amorgos (6%), Kythira (5%), Paros (5%), Tinos (5%), Syros (3%), Oinousses (2%), and Ios (2%). The mean age of study patients was 66 ± 8 years. More than half of them were women (60%) and married (80%). Most patients had cognitive complaints, while one-fifth of them were examined for a movement disorder (80% and 20%, respectively). The majority of caregivers and healthcare professionals were also women (72% and 91%, respectively), with a mean age of 61 ± 15 and 31 ± 7 years, respectively. Most caregivers and healthcare professionals that responded to the questionnaires were present during a telemedicine visit for a patient with cognitive complaints (72% and 91%, respectively). 

### 3.2. Perceptions toward the Use of Telemedicine

**Figure 1:**
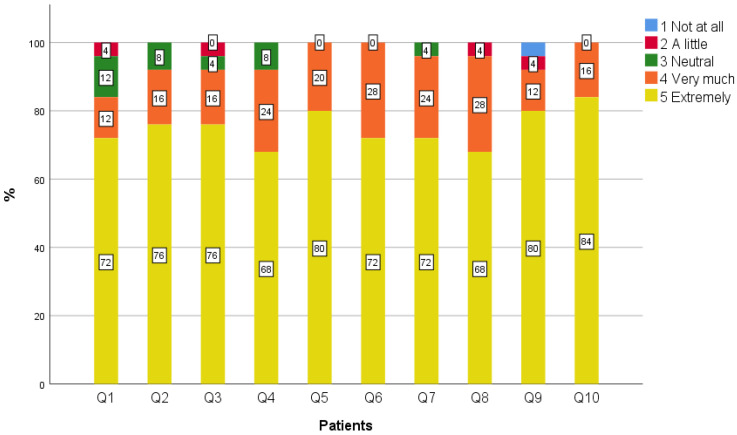
Perceptions of patients toward the use of the telemedicine service.

**Figure 2:**
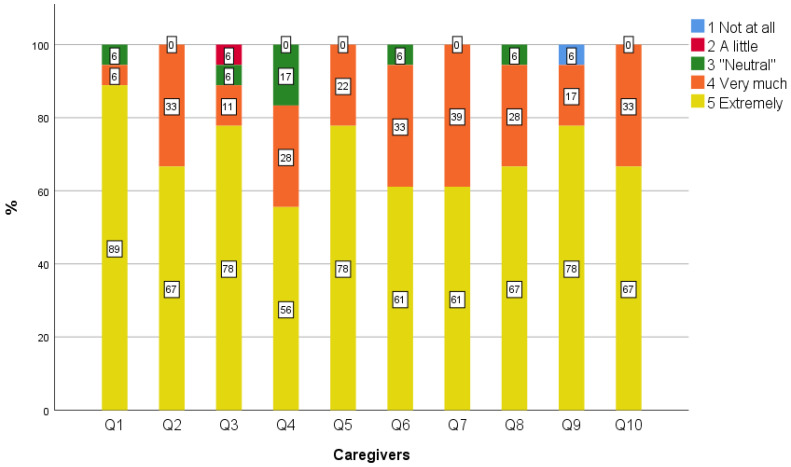
Perceptions of caregivers toward the use of the telemedicine service.

**Figure 3:**
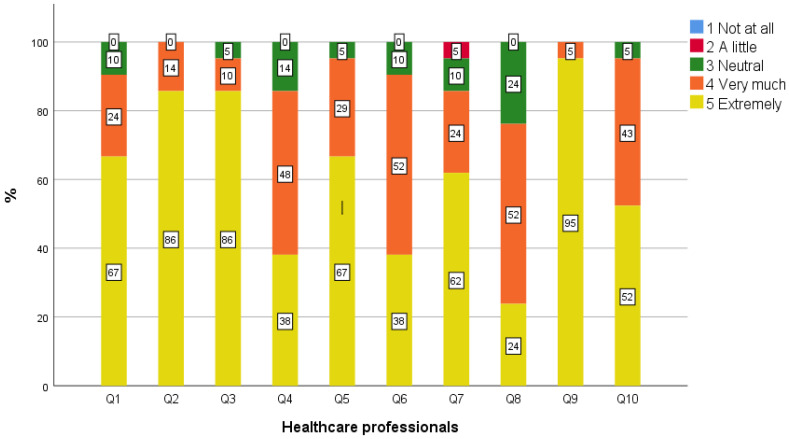
Perceptions of healthcare professionals toward the use of the telemedicine service.

Overall, all patients, caregivers, and the majority of healthcare professionals (95%) perceived the use of our telemedicine service positively, and they would select it again in the future (Table 3, Figure 1, Figure 2 and Figure 3). As for the specific aspects of telemedicine use, the highest scores were observed in the field of increased access to specialized care, since all caregivers and healthcare professionals and most patients (92%) agreed with this statement. Furthermore, reduced cost of medical follow-up and number of necessary transportations from permanent residency were mentioned by most respondents (92% patients, 94% caregivers, 100% healthcare professionals, and 92% patients, 89% caregivers, and 95% healthcare professionals, respectively). Most patients (92%) believed that telemedicine gives them the possibility for adequate medical follow-up, while this percentage was approximately 10% and 5% lower for caregivers and healthcare professionals, respectively.

A minority of patients (16%), caregivers (6%), and healthcare professionals (10%) mentioned that they were not as comfortable with talking to the doctors about medical problems through telemedicine as they would be if the examination was carried out in person. All patients, as well as most caregivers and healthcare professionals (94% and 90%, respectively), felt that the doctors made a reliable medical assessment through telemedicine, compared to if the examination was carried out in person. This score was 10% lower among healthcare professionals than among patients. Positive perceptions were expressed by most patients and caregivers (96% and 100%, respectively) and by a lower proportion of healthcare professionals (86%) regarding how efficiently the doctors explained the medical condition compared to if the examination was carried out in person. Moreover, although most participants agreed that the doctors significantly contributed to the improvement of the patients’ health status, these scores were 20% lower among healthcare professionals, compared to patients and caregivers (96% patients, 94% caregivers, 76% healthcare professionals). 

Healthcare professionals expressed statistically significantly lower scores compared to patients and caregivers in terms of the contribution of the telemedicine team to the improvement of the patients’ health status (*p* = 0.003). Healthcare professionals also displayed significantly lower scores in terms of the reliability of the telemedicine medical assessment, compared to patients and caregivers (*p* = 0.05). No significant differences were detected between the three subgroups (patients, caregivers, and healthcare professionals) regarding the other questions and the total score. Although not statistically significant, compared to patients and caregivers, lower scores were also observed among healthcare professionals on overall satisfaction, how efficiently the medical condition was explained, the reliability of the remote medical evaluation compared to a hypothetical in-person assessment with the telemedicine team, and the future selection of telemedicine. No differences were observed for the other survey statements between the different participant types.

No statistically significant differences were observed based on sex, educational level, marital status, reason for referral (cognitive or movement disorder), and island of residency (residency in Rhodes or not) for each question as well as the total score. Regarding the relationship between age and responses to the survey questions, there was a weak correlation, as investigated by the implementation of the non-parametric Spearman’s rho test, rho = 0.32, *p* = 0.014.

### 3.3. Participants’ Comments and Recommendations for the Improvement of the Telemedicine Service

In total, fourteen (56%) patients, six (33%) caregivers, and four (19%) healthcare professionals provided recommendations for the improvement of the telemedicine service at the end of the questionnaire (Table 4 and Table 5).

Among patients, the most common remark was the need for more frequent follow-up telemedicine visits, which was mentioned by approximately one-third of the respondents (36%). One patient also highlighted the need for raising awareness about the availability of the service (7%), and the need for more medical specialties (7%). Three patients mentioned that they did not have any specific recommendations for improvement (21%). Additional comments included that this specialized telemedicine service made them feel less isolated (14%), they could speak with someone that understands them (14%), they felt secure (7%), they could trust the doctors (14%), and that the medical assessment was correct (7%).

The availability of more medical specialties, the possibility of a private visit with the telemedicine doctors, as well as more frequent visits were the most common recommendations among caregivers (33% for each). The coverage of more remote areas was also mentioned (17%). One caregiver expressed his complaint about the fact that the telemedicine doctors did not call the patient back to ask for his/her health and see him/her again (17%). Another participant would like to meet the doctors additionally in person (17%). Better access to specialized care was reported as an advantage (17%).

Half of the healthcare professionals mentioned that it would be helpful if the telemedicine team could send a brief medical report on the examination findings to the referral doctors (50%). One healthcare professional suggested that the neuropsychological examination could be more individualized to the specific patients’ deficits (25%).

## 4. Discussion

This study shows positive perceptions among most patients, their caregivers, and to a lesser extent healthcare professionals living in the Aegean Islands toward the use of a specialized telemedicine service for cognitive and movement disorders. Most participants perceived all the different aspects of telemedicine positively, including the reliability of medical assessment (patients (Ps): 100%, caregivers (CGs): 94%, healthcare professionals (HPs): 90%), cost (Ps: 92%, CGs: 94%, HPs: 100%), number of transportations (Ps: 92%, CGs: 89%, HPs: 95%), improvement in health status (Ps: 96%, CGs: 94%, HPs: 76%), adequacy of follow-up (Ps: 92%, CGs: 83%, HPs: 86%), information delivery from the physicians (Ps: 96%, CGs: 100%, HPs: 86%), comfort (Ps: 84%, CGs: 94%, HPs: 90%), access to specialized care (Ps: 92%, CGs: 100%, HPs: 100%), and future selection of telemedicine (Ps: 100%, CGs: 100%, HPs: 95%).

In agreement with our results, previous studies have reported high satisfaction rates and positive perceptions toward telemedicine among patients with dementia and their caregivers in rural areas [3,9,10,11,34]. Mean scores of 4.67–4.84 are suggestive of comparable rates to other studies [11]. Regarding the various telemedicine aspects, patients and caregivers living in remote areas have been shown to be satisfied with remote telemedicine memory clinics in terms of reduction in travel distance, personal comfort, waiting time for the appointment, privacy, skillfulness of the telemedicine team, and explanation of treatment [3,35]. Another study also indicated that patients and caregivers agreed that the communication between patients and physicians was easy (mean 4.85 on a 5-point Likert scale), telemedicine saved travel time and cost (estimated round trip distance saved in miles, mean: 67.1, estimated round trip time on the road saved in minutes, mean: 74.5, estimated money saved on gasoline in USD, mean: 13.96), and they would use the telemedicine dementia service again (mean 4.58 on a 5-point Likert scale) [3]. Studies on Parkinson’s disease have demonstrated that patients positively perceive the use of telemedicine regarding convenience, improved accessibility, and reduced travel distance to medical assessment [7,36].

Compared to the number of studies on patients’ and caregivers’ perceptions, relevant evidence on the perceptions of healthcare professionals toward the use of telemedicine for neurodegenerative diseases is scant. The mean overall satisfaction of local healthcare professionals was 4.48, which is consistent with existing evidence [37]. High satisfaction has been expressed among local physicians with telepsychiatry for older adults in terms of convenience, positive impact on patients’ health, provision of sufficient medical information, and future use of telemedicine [11,34]. In line with this evidence, another study has shown that physicians agreed that telemedicine sessions at a rural memory disorder clinic assisted in clinical decision-making and enhanced the timely access to care [19].

Notably, healthcare professionals expressed significantly lower scores than patients and caregivers in terms of the contribution to improving health status as well as the reliability of the remote medical evaluation compared to a hypothetical in-person assessment by the telemedicine team. Although not statistically significant, lower scores were also observed among healthcare professionals on overall satisfaction, how efficiently the medical condition was explained, and future selection of telemedicine. In Greece, there is a lack of general practitioners and neurologists in rural areas, and medical workload is often too heavy, especially in the summer because of the tourist season. Although the NTN provides substantial organizational and administrative support for arranging and conducing the telemedicine examinations, additional time and work is often required from local physicians, including the completion of patients’ medical reports that are subsequently sent to the telemedicine team and participation in the telemedicine visit. Currently, there is no reimbursement or other incentives for the participation of local healthcare professionals in telemedicine services in Greece. Hence, their general lower scores compared to those of patients and caregivers might be explained by these organizational and financial aspects. In addition, the fact that there is still no definite cure for neurodegenerative diseases could be a potential reason for the relatively low scores in the question regarding the contribution of the telemedicine service to improving the patients’ health status. However, early diagnosis and appropriate symptomatic treatment provided by specialized care may significantly contribute to a better quality of life, which might be evaluated as a more important factor by the patients and caregivers compared to the healthcare professionals. Healthcare professionals are more aware of the limitations of neurological examination via video (e.g., rigidity, postural instability assessment) compared to patients and caregivers, which could potentially explain their lower scores in their perceptions of the reliability of video examination compared to a hypothetical in-person examination by the same medical team. This difference highlights the need for the development of standardized protocols for the remote examination of patients with cognitive and movement disorders, as well as further studies investigating the reliability of specific parts of neurological examination in virtual settings for this patient subpopulation.

The most common patient recommendations for the improvement of the telemedicine service were the need for more frequent visits (36%), followed by raising awareness about the availability of the service (7%) and the need for more medical specialties (7%). These comments further highlight the satisfaction of patients with specialized telemedicine services and their willingness to continue to use them. Additional remarks from caregivers included the need for a personal visit with the telemedicine team (33%) in order to further discuss their concerns about the patients in private. Local physicians also mentioned that it would be helpful if the telemedicine team could send a brief medical report on the examination findings to the referral doctors (50%). Although the telemedicine physicians informed the patients, caregivers, and healthcare professionals about the diagnosis and treatment plan, a written report should be also sent to the local physicians after the completion of the telemedicine evaluation. Since this is a pilot study, with the expansion of this activity, doctors dedicated to telemedicine will have more time to spend on telemedicine visits and fulfill that need. Furthermore, one healthcare professional suggested that the neuropsychological examination could be more individualized to the specific patients’ deficits (25%). However, given the limited duration of the telemedicine visit (approximately 1 h), there is usually no available time for additional neuropsychological testing at the initial assessment. This recommendation would be feasible during a second telemedicine visit.

Audio and visual clarity, as well as waiting time complaints, have been reported as reasons for dissatisfaction among patients in a community-based telepsychiatry service for older adults living in remote regions [11]. However, technical issues were not mentioned by the participants of our study, possibly due to the fact that the NTN provided the technological infrastructure, including high-resolution cameras and a relatively stable internet connection. Recommendations for reducing waiting times for telemedicine appointments were not mentioned by our respondents. One probable reason could be the fact that waiting times for in-person specialized neurological evaluation are often too long in our country, while the mean waiting time for the telemedicine visit was approximately one week. Furthermore, in contrast to our results, another study demonstrated that general practitioners were dissatisfied with the referral process as too much information was needed [11]. Better patient–physician communication and network connection, affordability, as well as a more organized delivery of telemedicine services have been mentioned as recommendations for the improvement of telemedicine in other patient populations [23]. The provision of telemedicine services in Greece via the NTN does not require out-of-pocket expenses from patients; hence, affordability was not mentioned as an issue in our case.

An important aspect that should be also mentioned is the fact that it is not easy for older individuals to accept and adopt new technologies. In this context, a recent study has demonstrated that most older patients receive computer support from their children and grandchildren, about two-thirds “were unfamiliar with the “Copy Paste” function”, while only one-third of them “were open to new ways of using computers” [38]. Furthermore, digital literacy has been associated with increased frequency of internet use, and also with lower levels of computer stress [38]. Another study conducted on older individuals during the COVID-19 pandemic also showed that those who had no internet access and had not participated in videoconferencing prior to the pandemic were less likely to mention having access to telehealth [39]. The presence of comorbidities was also associated with higher rates of telehealth use when offered in that study [39]. Optimism and innovativeness have been associated with perceived ease of use of digital health technologies among older adults [40]. Our telemedicine model did not require patients to use a computer, the internet, or other devices, since local healthcare professionals with the assistance of the NTN HelpDesk coordinated the whole process of remote examination, including the arrangement of appointments and videoconferencing. Nevertheless, it would be interesting for future studies to investigate the factors that may affect the acceptability and adoption of this kind of telemedicine services among older patients with cognitive and movement disorders.

This study is one of the first systematic attempts to identify the perceptions of patients, caregivers, and healthcare professionals regarding the use of telemedicine services provided by a tertiary academic center in Greece. The positive responses of most participants suggest that this model of telemedical care might be feasible and acceptable in the Greek population in remote or rural areas of the mainland too, and used by other medical specialties, such as psychiatry, endocrinology, rheumatology, etc., and other neurological disorders, such as headache, epilepsy, etc. Furthermore, the technological requirements for this kind of telemedicine service do not include any sophisticated devices, except for a high-resolution camera and screen as well as a stable and safe internet connection, which are relatively easily adaptable to diverse healthcare settings. In addition, the patients and caregivers were not expected to learn to use the technological equipment, since the healthcare provider facilitated the whole remote assessment process locally. The participation of patients in this telemedicine service also did not require any out-of-pocket costs. However, the NTN played a pivotal role in the whole process via its significant administrative and organizational contribution. Hence, the results of our study could be applicable in other countries too, where a similar telemedicine network exists at a regional or national level and no additional out-of-pocket costs are required from the patients. The limited availability of neurologists in remote and rural areas is a universal phenomenon, while the prevalence of dementia and movement disorders will continue to grow [41,42]. In general, these results agree with existing literature evidence on the perceptions of participants toward telemedicine services in other geographical locations and healthcare settings [2], further supporting the assumption that these findings are not unique to the Aegean Islands. Hence, the provision of specialized care via telemedicine, particularly for remote and underserved areas, could be an effective solution. Given the relatively low number of total telemedicine evaluations during this 2-year period, as well as the patients’ comments that more individuals need to know about the availability of this service, attempts should be made towards the promotion of this service and outreach to patients but also to local doctors, nurses, and other community healthcare professionals. Effective collaboration with local municipalities and other communities and patients’ associations could significantly aid in this process.

It can be also hypothesized that patients who were more willing to be assessed through telemedicine would also have perceived this means of care more positively compared to patients who had refused to use this service. Therefore, our results can be generalized to patients who are willing to use this service when it is offered to them. It would be interesting for future research to investigate the relationship between the willingness to use telemedicine and satisfaction with its use.

The overall acceptability of a service or an application by the end-users can be referred to as quality of experience (QoE), which also depends on the quality of service (QoS) that is provided by the underlying communication systems [43,44]. From a QoS point of view, telemedicine services need to be available, reliable, and also guarantee low delay, while data security and profitability constitute additional QoS features. Evaluation approaches for QoE can vary and may for instance include recording user perceptions during a telemedicine session involving audiovisual communication with healthcare experts. In this regard, a qualitative and quantitative study aiming to evaluate the network QoS performance and end-user QoE perception of telemedicine systems in Mali and Senegal using the publicly accessible conversation tool Skype demonstrated that about three-quarters of the end-users were satisfied with the quality of the telemedicine service, and about two-thirds of the end-users had a positive impression of the network’s performance [45]. Based on these results, a mathematical formula was also proposed to predict the corresponding QoE, given the existing relationship between QoS and QoE. Since this is a pilot study, future research could focus on QoS and QoE in the NTN and our telemedicine service model in particular in order to examine patients’ and caregivers’ perceptions in a more systematic way.

Our study has several limitations. First, the response rate was relatively low. However, response rates in relative studies on satisfaction and perceptions on telemedicine for dementia are also relatively low, ranging from 2.8 to 88% [46]. It is considered that participants who are extremely satisfied or dissatisfied are more likely to respond to such surveys; hence, it could be speculated that missing questionnaires might be rather “neutral” [47]. Nevertheless, given the fact that this study is one of the first attempts to describe the perceptions of patients, caregivers, and healthcare professionals toward specialized telemedicine services in Greece, the results of our study provide useful information and insights despite the relatively low response rates. Moreover, there was an unequal distribution of the islands of residency among the respondents, since approximately half of the telemedicine visits and three-quarters of the respondents were from Rhodes. The varied interest and engagement of peripheral general practitioners in this newly designed telemedicine service could be a potential reason. The service of the “Specialized Outpatient Clinic of Memory, Dementia and Parkinson’s disease through the National Telemedicine Network”, including the telemedicine team providing remote neurological, psychiatric, and neuropsychological assessments, was financially supported by the Interreg ADRION Program funded by the European Regional Development Fund and IPA II fund (Project Number 1228). However, there was no particular reimbursement for local healthcare professionals on the islands who participated in the telemedicine visit, and their involvement in this program was incorporated into their regular clinical services in a volunteer basis, without being “obliged” to get involved. Hence, local healthcare professionals who are more willing and enthusiastic about telemedicine were probably more likely to participate, possibly contributing to selection bias. Another possible limitation is the fact that the vast majority of local healthcare providers were women. In this context, it has been previously demonstrated that female doctors may be more likely to not only use telemedicine services, but also to perceive the benefits of telemedicine compared to male doctors [48], which may also at least partially explain the gender distribution of healthcare providers in our study.

Furthermore, the inhabitants of remote areas of Greece, including the islands, do not have adequate access to specialized care. Hence, the high scores in our study might be at least partially attributed to the opportunity of these patients to receive specialized neurological, psychiatric, and neuropsychological assessment, and not their experience with the telemedicine service itself. Potential selection bias should be also considered, since complicated or difficult cases with ambiguous diagnosis and/or ineffective management would be more likely to be referred to this specialized telemedicine unit. Concerning generalizability, it should be noted that this telemedicine service is integrated within the NTN, which is the most well-established telemedicine network in Greece and which provided the infrastructure (technological equipment, administrative support, scheduling of appointments, etc.). Another limitation is the fact that some other aspects of telemedicine use, such as ethical considerations, data privacy, or preference for in-person over telemedicine visits, were not included in the questionnaires. Our survey was intended to be easy to complete, but it would be useful to assess these additional elements in future studies. In addition, we could not investigate if there were differences in perceptions between first and follow-up visits, since participants were asked to fill in the surveys after the initial assessment. Deeper qualitative analysis via conducting semi-structured interviews would also probably provide us with more insightful evidence regarding the perceptions of participants on telemedicine. We are going to continue this research work by performing semi-structured interviews with a representative sample from our study as part of a future research project of our team. Furthermore, it would be of interest for future studies to estimate the proportion of patients living in regions without an available neurologist, since this information would help us identify the areas and number of patients needing this service the most.

Despite these limitations, our study demonstrates, for the first time in the Greek population, that most patients with cognitive and movement disorders, their caregivers, and to a lesser extent healthcare professionals in the Aegean Islands positively perceive the use of this specialized telemedicine service. It is one of few studies that concurrently investigate the perceptions of patients, caregivers, and healthcare professionals, as well as their recommendations for the improvement of the telemedicine service. This evidence further contributes to the existing literature, provides insights for the optimization of telemedicine for patients with neurodegenerative disorders, and highlights the value of telemedicine for equal healthcare provision, especially in remote, underserved regions beyond the COVID-19 pandemic.

## 5. Conclusions

In conclusion, telemedicine seems to constitute a valuable tool for the provision of care for cognitive and movement disorders in the Aegean Islands by overcoming geographical barriers and improving the access of patients living in remote and rural areas to specialized healthcare. Our research team’s aim is to continue to improve this specialized telemedicine service based on patients’, caregivers’, and healthcare professionals’ feedback, with the ultimate goal of optimizing clinical outcomes.

## Figures and Tables

**Table 1:** Characteristics of patients examined via telemedicine at the “Specialized Outpatient Clinic of Memory, Dementia and Parkinson’s disease through the National Telemedicine Network” during the period March 2021–March 2023.

Characteristics	Patients (n = 68)
Age (mean, SD)	68 (11.4)
Female sex (n, %)	42 (62%)
Island of residency	
Rhodes (n, %)	37 (54.4%)
Amorgos (n, %)	3 (4.4%)
Tinos (n, %)	3 (4.4%)
Syros (n, %)	1 (1.5%)
Paros (n,%)	3 (4.4%)
Ios (n, %)	4 (5.9%)
Kythira (n, %)	4 (5.9%)
Oinousses (n, %)	1 (1.5%)
Agios Efstratios (n, %)	1 (1.5%)
Astypalaia (n, %)	3 (4.4%)
Folegandros (n, %)	1 (1.5%)
Kalymnos (n, %)	1 (1.5%)
Kimolos (n, %)	3 (4.4%)
Kos (n, %)	1 (1.5%)
Patmos (n, %)	1 (1.5%)
Samos (n, %)	1 (1.5%)
Education years (mean, SD)	10 (4.6)
Marital status	
Single (n, %)	5 (7%)
Married (n, %)	42 (62%)
Divorced (n, %)	3 (4%)
Widowed (n, %)	13 (19%
Unknown (n,%)	5 (7%)
Reason of referral	
Cognitive disorder (n, %)	57 (84%)
Movement disorder (n, %)	10 (15%)
Other (n, %)	1 (1.5%)

**Table 2:** Demographic characteristics of study participants regarding perceptions toward telemedicine use (patients, caregivers, healthcare professionals) and reason for referral for telemedicine examination.

Demographic Characteristic	Patients	Caregivers	Healthcare Professionals
N, %	25/64 (39%)	18/64 (28%)	21/64 (33%)
Mean age in years (SD)	66 (8)	61 (15)	31 (7)
Female sex (n, %)	15 (60%)	13 (72%)	19 (91%)
Island of residency			
Rhodes (n, %)	17 (68%)	11 (61%)	19 (91%)
Amorgos (n, %)	2 (8%)	2 (11%)	0 (0%)
Tinos (n, %)	1 (4%)	1 (6%)	1 (5%)
Syros (n, %)	1 (4%)	1 (6%)	0 (0%)
Paros (n,%)	2 (8%)	0 (0%)	1 (5%)
Ios (n, %)	0 (0%)	1 (6%)	0 (0%)
Kythira (n, %)	1 (4%)	2 (11%)	0 (0%)
Oinousses (n, %)	1 (4%)	0 (0%)	0 (0%)
Marital status			
Single (n, %)	1 (4%)	3 (17%)	14 (67%)
Married (n, %)	20 (80%)	14 (78%)	7 (33%)
Divorced (n, %)	2 (8%)	0 (0%)	0 (0%)
Widowed (n, %)	2 (8%)	1 (6%)	0 (0%)
Highest educational attainment			
Primary (n, %)	6 (26%)	5 (29%)	0 (0%)
Secondary (n, %)	8 (35%)	7 (41%)	0 (0%)
Tertiary (n, %)	8 (35%)	5 (29%)	16 (76%)
Master’s or doctoral degree (n, %)	1 (4%)	0 (0%)	5 (24%)
Reason for referral			
Cognitive disorder (n, %)	20 (80%)	13 (72%)	19 (91%)
Movement disorder (n, %)	5 (20%)	5 (28%)	2 (10%)

**Table 3:** Perceptions of patients, caregivers, and healthcare professionals toward telemedicine use.

Survey Statements (Telemedicine Aspects)	Not at All (n, %)	A Little (n, %)	Neutral (n, %)	Very Much (n, %)	Extremely (n, %)	Positive Response (Very Much or Extremely, n, %)	Mean (SD)	Median (IQR)	*p*-Value *
Q1. “I felt comfortable talking to the doctors about medical problems through telemedicine, as if the conversation was carried out in-person”									
Patient	0 (0%)	1 (4%)	3 (12%)	3 (12%)	18 (72%)	21 (84%)	4.5 (0.9)	5 (1)	*p* = 0.29
Caregiver	0 (0%)	0 (0%)	1 (6%)	1 (6%)	16 (89%)	17 (94%)	4.8 (0.5)	5 (0)	
Healthcare professional	0 (0%)	0 (0%)	2 (10%)	5 (24%)	14 (67%)	19 (90%)	4.6 (0.7)	5 (1)	
Q2. “Telemedicine helped in having increased access to specialized healthcare”									
Patient	0 (0%)	0 (0%)	2 (8%)	4 (16%)	19 (76%)	23 (92%)	4.7 (0.6)	5 (1)	*p* = 0.40
Caregiver	0 (0%)	0 (0%)	0 (0%)	6 (33%)	12 (67%)	18 (100%)	4.7 (0.5)	5 (1)	
Healthcare professional	0 (0%)	0 (0%)	0 (0%)	3 (14%)	18 (86%)	21 (100%)	4.9 (0.4)	5 (0)	
Q3. “Because of telemedicine, transportations were reduced from the permanent residency”									
Patient	0 (0%)	1 (4%)	1 (4%)	4 (16%)	19 (76%)	23 (92%)	4.6 (0.8)	5 (1)	*p* = 0.69
Caregiver	0 (0%)	1 (6%)	1 (6%)	2 (11%)	14 (78%)	16 (89%)	4.6 (0.9)	5 (0)	
Healthcare professional	0 (0%)	0 (0%)	1 (5%)	2 (10%)	18 (86%)	20 (95%)	4.8 (0.5)	5 (0)	
Q4. “Telemedicine gives the possibility for adequate follow-up”									
Patient	0 (0%)	0 (0%)	2 (8%)	6 (24%)	17 (68%)	23 (92%)	4.6 (0.7)	5 (1)	*p* = 0.17
Caregiver	0 (0%)	0 (0%)	3 (17%)	5 (28%)	10 (56%)	15 (83%)	4.4 (0.8)	5 (1)	
Healthcare professional	0 (0%)	0 (0%)	3 (14%)	10 (48%)	8 (38%)	18 (86%)	4.2 (0.7)	4 (1)	
Q5. “I would select the examination through telemedicine again in the future”									
Patient	0 (0%)	0 (0%)	0 (0%)	5 (20%)	20 (80%)	25 (100%)	4.8 (0.4)	5 (0)	*p* = 0.51
Caregiver	0 (0%)	0 (0%)	0 (0%)	4 (22%)	14 (78%)	18 (100%)	4.8 (0.4)	5 (0)	
Healthcare professional	0 (0%)	0 (0%)	1 (5%)	6 (29%)	14 (67%)	20 (95%)	4.6 (0.6)	5 (1)	
Q6. “The doctors made a reliable medical assessment through telemedicine, as if the examination was carried out in-person”									
Patient	0 (0%)	0 (0%)	0 (0%)	7 (28%)	18 (72%)	25 (100%)	4.7 (0.5)	5 (1)	*p* = 0.05 **
Caregiver	0 (0%)	0 (0%)	1 (6%)	6 (33%)	11 (61%)	17 (94%)	4.6 (0.6)	5 (1)	
Healthcare professional	0 (0%)	0 (0%)	2 (10%)	11 (52%)	8 (38%)	19 (90%)	4.3 (0.6)	4 (1)	
Q7. “The doctors efficiently explained the medical condition as if the examination was in-person”									
Patient	0 (0%)	0 (0%)	1 (4%)	6 (24%)	18 (72%)	24 (96%)	4.7 (0.6)	5 (1)	*p* = 0.64
Caregiver	0 (0%)	0 (0%)	0 (0%)	7 (39%)	11 (61%)	18 (100%)	4.6 (0.5)	5 (1)	
Healthcare professional	0 (0%)	1 (5%)	2 (10%)	5 (24%)	13 (62%)	18 (86%)	4.4 (0.9)	5 (1)	
Q8. “The doctors significantly contributed to the improvement of health status”									
Patient	0 (0%)	1 (4%)	0 (0%)	7 (28%)	17 (68%)	24 (96%)	4.6 (0.7)	5 (1)	*p* = 0.003 **
Caregiver	0 (0%)	0 (0%)	1 (6%)	5 (28%)	12 (67%)	17 (94%)	4.6 (0.6)	5 (1)	
Healthcare professional	0 (0%)	0 (0%)	5 (24%)	11 (52%)	5 (24%)	16 (76%)	4.0 (0.7)	4 (1)	
Q9. “Telemedicine reduced the cost of the medical follow-up”									
Patient	1 (4%)	1 (4%)	0 (0%)	3 (12%)	20 (80%)	23 (92%)	4.6 (1)	5 (0)	*p* = 0.24
Caregiver	1 (6%)	0 (0%)	0 (0%)	3 (17%)	14 (78%)	17 (94%)	4.6 (1)	5 (0)	
Healthcare professional	0 (0%)	0 (0%)	0 (0%)	1 (5%)	20 (95%)	21 (100%)	5.0 (0.2)	5 (0)	
Q10. “I am generally satisfied with the telemedicine examination”									
Patient	0 (0%)	0 (0%)	0 (0%)	4 (16%)	21 (84%)	25 (100%)	4.8 (0.4)	5 (0)	*p* = 0.06
Caregiver	0 (0%)	0 (0%)	0 (0%)	6 (33%)	12 (67%)	18 (100%)	4.7 (0.5)	5 (1)	
Healthcare professional	0 (0%)	0 (0%)	1 (5%)	9 (43%)	11 (52%)	20 (95%)	4.5 (0.6)	5 (1)	
Total score									
Patient	-	-	-	-	-	-	36.7 (4.2)	40 (7)	*p* = 0.34
Caregiver	-	-	-	-	-	-	36.3 (3.8)	37.5 (8.3)	
Healthcare professional	-	-	-	-	-	-	35.2 (4.1)	36 (4)	

* Kruskal–Wallis H test. ** Statistically significant.

**Table 4:** Recommendations for the improvement of the telemedicine service expressed by patients, caregivers, and healthcare professionals.

Participant Types	Recommendations for the Improvement of the Telemedicine Service
Patients	“I don’t have a specific recommendation. I am generally very satisfied with the exam via telemedicine. I hope it would be available for more medical specialties” “I would like more frequent follow-up. For the first time someone dealt with me so much” “Thank you very much. There was an opportunity to talk and somebody to understand me” “I want to solve my medical problem. Nobody has helped me till now” “I would like more frequent telemedicine visits” “I would like more frequent visits. We did not feel uncomfortable. We felt that something can be done for us in the island remotely. Your assessment was right and I felt I can trust the doctors” “I don’t have significant recommendations for improvement. The lower rating in some fields is attributed to the nature of the service, and I don’t know if it can be improved” “I am very satisfied with the examination. I can’t think about any recommendation for improvement” “I would like more frequent follow-up, at least twice a year” “Please continue your excellent work” “I suggest frequent and regular follow-up, because I trust you and I feel secure via this way” “More people need to be aware of your telemedicine clinic. Very important help. I feel that somebody understands me” “Thank you very much. I feel that I am not isolated with this way” “I am very satisfied”
Caregivers	“I would like more frequent visits and follow-up. I felt very comfortable talking to the doctors about the problem of my husband. Telemedicine is a very good solution, but I would also like to meet the doctors in-person at the hospital if possible” “I would like to have a personal appointment with the medical doctors of the telemedicine visit, in order to ask some queries and inform about some situations in private” “For us who live in remote areas and we have only the health center, it is very important to receive specialized examination. I would like more frequent assessment in order to be fully informed, and more medical specialties. But, no one asked me if the health condition was improved. I would like for you to ask us to see us again” “I hope all people take advantage of the provision of telemedicine, in order for their medical conditions to be solved. I wish telemedicine would be possible for more medical specialties, and for more remote regions” “Your work is excellent” “I would like to have the possibility to talk about my mother and her memory problems in private, in order to be more helpful in what I have to be careful for and observe in her behavior in general”
Healthcare professionals	“I have no specific recommendation for improvement. There is enough space in the room. It is very helpful that the medical instructions are sent via e-mail to us” “It would be helpful if a brief medical report could be electronically sent to the healthcare professionals that made the referral, regarding the conclusions of the examination” “A brief report with the results of the visit would be helpful to be sent to the healthcare professionals” “It would be helpful if the neuropsychological assessment was more individualized for the patient’s deficits”

**Table 5:** Frequency of comments and recommendations for the improvement of the telemedicine service expressed by patients, caregivers, and healthcare professionals.

Participant Types	Frequencies of Comments and Recommendations for the Improvement of the Telemedicine Service
Patients	Need for more frequent follow-up telemedicine visits (36%) No specific recommendations for improvement mentioned (21%) Need to raise awareness about the availability of the service (7%) Need for more medical specialties (7%) This specialized telemedicine service made them feel less isolated (14%) Opportunity to speak with someone that understands them (14%) Feeling secure (7%) Trusting the doctors (14%) The medical assessment was correct (7%)
Caregivers	Need for more medical specialties (33%) Need for a private visit with the telemedicine doctors (33%) Need for more frequent visits (33%) Need for coverage of more remote areas (17%)
	Complaining about the fact that the telemedicine doctors did not call the patient back to ask for his/her health and see him/her again (17%) Need for meeting the telemedicine doctors additionally in person (17%) Better access to specialized care (17%)
Healthcare professionals	It would be helpful if the telemedicine team could send a brief medical report on the examination findings to the referral doctors (50%)
	The neuropsychological examination could be more individualized to the specific patients’ deficits (25%)

## Data Availability

Data are available on request due to restrictions, e.g., privacy or ethics.
